# Tubulointerstitial nephritis with uveitis syndrome: A case report and review of literature

**DOI:** 10.4103/0971-4065.65307

**Published:** 2010-04

**Authors:** S. Parameswaran, N. Mittal, K. Joshi, M. Rathi, H. S. Kohli, V. Jha, K. L. Gupta, V. Sakhuja

**Affiliations:** Department of Nephrology, PGIMER, Chandigarh, India; 1 Pathology, PGIMER, Chandigarh, India

**Keywords:** Acute interstitial nephritis, TINU syndrome, uveitis

## Abstract

Tubulointerstitial nephritis with uveitis (TINU) syndrome is an unusual and under diagnosed cause of acute interstitial nephritis. The interstitial nephritis may precede, follow or develop concurrent to the uveitis. About 200 cases have been reported worldwide with only a single case reported from India. We report a 16-year-old male with TINU syndrome.

## Introduction

Acute tubulointerstitial nephritis (AIN) is usually related to medication or infection. AIN may also develop as part of a systemic illness but the distinct entity of tubulointerstitial nephritis with uveitis (TINU) syndrome is an uncommon cause of AIN. More than 200 cases have been reported worldwide but we could find only one case reported from India. We report a case of AIN who developed bilateral anterior uveitis on follow-up.

## Case Report

A 16-year-old male presented with hypertension, prolonged fever and impaired renal function. Three months prior to presentation he developed headache and was detected to have high blood pressure. No further evaluation was undertaken and no anti-hypertensives were prescribed. Three weeks before presentation he developed fever up to 100°F. The fever persisted despite a course of chloroquine and ciprofloxacin. During evaluation of the fever he was detected to have elevated serum creatinine and was referred to us. He had no urinary symptoms other than nocturia, which was first noticed two months back. He had no history of tuberculosis in the past, neither was there any family history of the same. He had no addictions and denied intake of analgesics or any indigenous medicine. His appetite was good and there was no recent weight loss. His blood pressure was 150/90 mmHg. All the peripheral pulses were normal and equally palpable. The physical examination was essentially normal; he had no edema or palpable lymph nodes. He had no abdominal bruit.

His hemoglobin was 12 gm/dl, TLC 6800/cumm and ESR 20 mm/1^st^ hour. His blood urea was 64 mg/dl and serum creatinine was stable at 3 mg/dl during the hospital stay. The urine examination revealed protein (+), glycosuria in the presence of normoglycemia and 15 to 20 WBC/HPF. The 24-hour urine protein excretion was 0.7 gm. The urine culture was repeatedly sterile. Urinalysis did not show presence of eosinophils or acid fast bacilli. Ultrasonography of the abdomen did not reveal any abnormality and showed a right kidney of 11.7cms and left kidney of 12 cms size. The liver function tests, serum calcium, uric acid and inorganic phosphate levels were all normal. A contrast enhanced CT scan of the chest and abdomen was performed and was normal.

**Figure 1:**
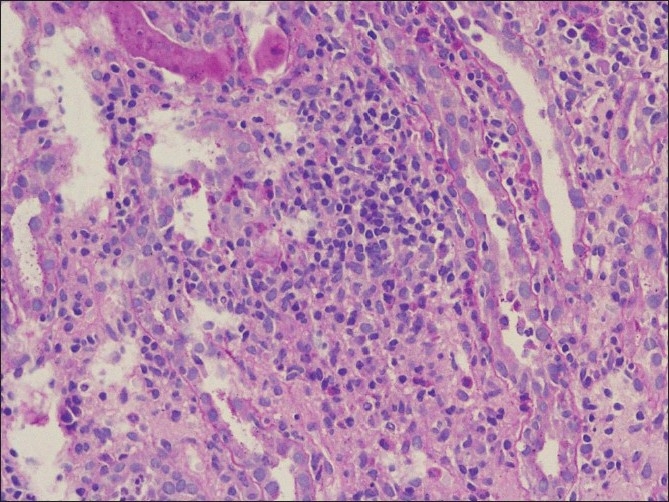
The interstitium is markedly expanded by lymphomononuclear inflammatory cells which are infiltrating the tubules with focal rupture (arrow) of the tubular basement membrane (PAS, ×200)

**Figure 2:**
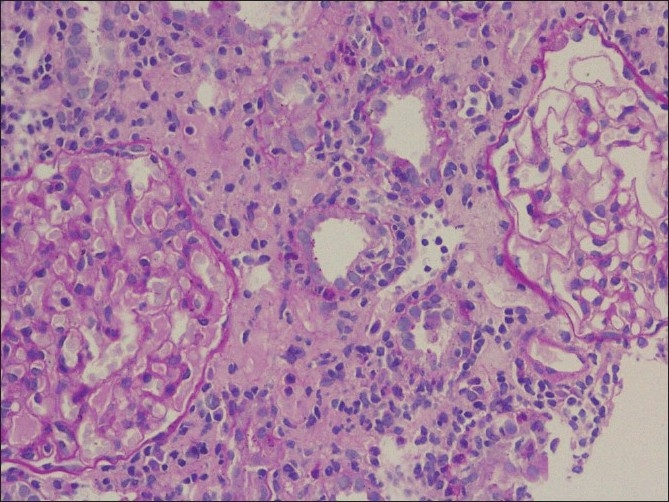
A focus of moderate tubulitis (arrow) along with moderate interstitial edema and inflammation is seen. In addition, the two glomeruli seen in the picture are within normal limits (PAS, ×200)

Antinuclear antibody, rheumatoid arthritis factor and anti neutrophil cytoplasmic antibody against myeloperoxidase and proteinase 3 by ELISA were negative. A provisional diagnosis of acute interstitial nephritis was made and the same was confirmed on a kidney biopsy [Figures 1 and 2]. Keeping the possibility of drug induced interstitial nephritis he was started on oral prednisolone 1mg/kg/day. He became afebrile and the serum creatinine came down to 1.3 mg/dl in two weeks. The steroid was tapered and stopped after six weeks. One month after discontinuation of prednisolone, he developed redness of both eyes without visual impairment or pain. An ophthalmology evaluation confirmed bilateral anterior uveitis and he was started on betamethasone and homatropine eye drops. A Schirmer’s test performed was normal. In the presence of biopsy documented acute interstitial nephritis and anterior uveitis in the absence of an obvious cause for both, a diagnosis of TINU syndrome was made. He remained asymptomatic for nine months and subsequently developed fever upto 100°F. Since fever persisted for more than two weeks without any localizing signs, he was given a short course of low dose steroids after which he became afebrile.

## Discussion

The entity of tubulointerstitial nephritis with uveitis (TINU syndrome) was first described by Dobrin *et al*. in 1975.[1] More than 200 cases have been reported worldwide,[2] mostly in the ophthalmology and pediatric medical literature. The mean age of presentation is 15 years (range 9 to 74years) and there is a female preponderance with earlier reports showing a female to male ratio of 3:1.[3] The proportion of males has been reported to be increasing in more recent reports.[2]

The clinical presentation is variable and has been comprehensively reviewed by Mandeville *et al*.[3] The interstitial nephritis precedes the uveitis in 65% of cases but follows ocular manifestations in 21% of cases and develops concurrent to uveitis in 15%. The most common initial symptoms are fever, weight loss, fatigue and malaise. Other systemic symptoms include anorexia, abdominal or flank pain, arthralgias, myalgias and headache. Lymphadenopathy, rash, facial edema and sore throat occur in less than 1% of cases. The renal manifestations include nocturia and polyuria (8%), sterile pyuria (55%), microhematuria (42%), subnephrotic proteinuria, tubular defects including renal glycosuria and impaired renal function. The serum creatinine is abnormal in at least 90% of patients with 64% of them having a creatinine >2 mg/dl. The ocular manifestation is in the form of anterior uveitis in 80% of patients but may also manifest as posterior or pan uveitis. The most common ocular symptoms are eye pain and redness (77%) and visual impairment occur in only 20%, usually in the presence of posterior uveitis.

Uveitis may develop up to two months before or up to 14 months after onset of systemic symptoms. When the ocular symptoms develop after systemic symptoms, the median time interval between the two was three months. Uveitis may recur in about 41% and the recurrence may occur up to two years after the first bout, but usually uveitis recurs within three months of discontinuation of steroids. In general, the course of ocular disease is independent from that of the renal disease. A few patients have been reported to have persistent mild renal insufficiency and only five patients have had renal failure severe enough to necessitate dialysis, of which two patients required long term dialysis.

The diagnosis of TINU syndrome is one of exclusion, based on the presence of uveitis and findings of acute interstitial nephritis in the absence of other disease states which can cause both uveitis and tubulointerstitial nephritis. Mandeville *et al*. has proposed diagnostic criteria for TINU syndrome [Table 1]. Diseases which can manifest acute interstitial nephritis along with ocular abnormalities need to be considered in the differential diagnosis of TINU syndrome and include Sjogren’s syndrome, sarcoidosis, tuberculosis and toxoplasmosis.

**Table 1:** Diagnostic criteria for TINU syndrome

The diagosis of TINU syndrome requires the presence of both AIN and uveitis without other known systemic disease that cause either interstitial nephritis or uveitis
Definite TINU syndrome:
AIN diagnosed histopathologically or clinically
(complete criteria*) and typical uveitis
Probable TINU syndrome:
AIN diagnosed histopathologically and atypical uveitis
or
AIN diagnosed clinically (incomplete criteria) and typical uveitis
Possible TINU syndrome:
AIN diagnosed clinically (incomplete criteria) and atypical uveitis

* The three clinical criteria required to make a clinical diagnosis of AIN are: (1) Abnormal renal function, (2) Abnormal urinalysis and, (3) A systemic illness lasting two weeks or more

β_2_ microglobulin and Krebs von den Lunge – 6 (KL-6) protein have been reported as two potential diagnostic markers in TINU syndrome. Urinary β_2_ microglobulin level, a marker of interstitial nephritis, was markedly elevated in almost every case tested[4,5] and may remain elevated for months after the urinalysis and serum creatinine have returned to normal. It may be useful in cases where renal biopsy is not indicated. Krebs von den Lunge-6 (KL-6) is a glycoprotein whose serum concentrations rise in response to various respiratory pathologies.[6] Compared to patients with uveitis from other causes, serum KL-6 levels in patients with TINU syndrome were significantly elevated and on renal biopsy the distal tubules of the patients with TINU syndrome stained strongly with anti-KL-6 antibody suggesting that the elevated KL-6 levels reflect the underlying renal lesion.[7] Serum KL-6 levels may prove to be a valuable tool in the diagnosis and follow-up of patients with TINU syndrome, especially if studies show a significant difference between patients with TINU syndrome compared with acute interstitial nephritis of other etiology.

The acute interstitial nephritis of TINU syndrome may resolve spontaneously and systemic corticosteroids are reserved for cases with persistent or progressive renal failure.[3] The anterior uveitis of TINU syndrome has been treated most commonly with topical corticosteroids and cycloplegic agents. There have been no prospective, randomized trials comparing steroid therapy with placebo, or addressing the optimum dose and duration of treatment. Uveitis in the setting of TINU syndrome appears to be more persistent and troublesome than the nephritis.[3] Immunomodulatory chemotherapeutic agents may be used when uveitis is unresponsive to systemic steroids or to reduce ocular or systemic toxicity from corticosteroids. The agents used include azathioprine, methotrexate, cyclosporine and mycophenolate mofetil.[3]
